# Two new asexual genera and six new asexual species in the family Microthyriaceae (Dothideomycetes, Ascomycota) from China

**DOI:** 10.3897/mycokeys.85.70829

**Published:** 2021-11-29

**Authors:** Min Qiao, Hua Zheng, Ji-Shu Guo, Rafael F. Castañeda-Ruiz, Jian-Ping Xu, Jie Peng, Ke-Qin Zhang, Ze-Fen Yu

**Affiliations:** 1 Laboratory for Conservation and Utilization of Bio-resources, Key Laboratory for Microbial Resources of the Ministry of Education, Yunnan University, Kunming, Yunnan, 650091, China; 2 School of Life Sciences, Yunnan University, Kunming, Yunnan, 650091, China; 3 Instituto de Investigaciones Fundamentales en Agricultura Tropical “Alejandro de Humboldt” (INIFAT), 17200, La Habana, Cuba; 4 Department of Biology, McMaster University, Hamilton, Ontario, L8S 4K1, Canada

**Keywords:** Aquatic hyphomycetes, asexual genera, Microthyriaceae, phylogeny

## Abstract

The family Microthyriaceae is represented by relatively few mycelial cultures and DNA sequences; as a result, the taxonomy and classification of this group of organisms remain poorly understood. During the investigation of the diversity of aquatic hyphomycetes from southern China, several isolates were collected. These isolates were cultured and sequenced and a BLAST search of its LSU sequences against data in GenBank revealed that the closest related taxa are in the genus *Microthyrium*. Phylogenetic analyses, based on the combined sequence data from the internal transcribed spacers (ITS) and the large subunit (LSU), revealed that these isolates represent eight new taxa in Microthyriaceae, including two new genera, *Antidactylaria***gen. nov.** and *Isthmomyces***gen. nov.** and six new species, *Antidactylariaminifimbriata***sp. nov.**, *Isthmomycesoxysporus***sp. nov.**, *I.dissimilis***sp. nov.**, *I.macrosporus***sp. nov.**, *Triscelophorusanisopterioideus***sp. nov.** and *T.sinensis***sp. nov**. These new taxa are described, illustrated for their morphologies and compared with similar taxa. In addition, two new combinations are proposed in this family.

## Introduction

The family Microthyriaceae (Microthyriales, Dothideomycetes) was established by Saccardo (1883), containing foliar epiphytes and saprobes on dead leaves and stems (Wu et al. 2011a). This family is characterised by having superficial, flattened thyriothecia, with cells of the upper wall radiating in a parallel arrangement from the central ostiole opening; the ostiole may or may not be surrounded by setae. Asci are fusiform or obclavate to cylindro-clavate, bitunicate and fissitunicate and ascospores are two-celled, hyaline to brown often with ciliate appendages (Ashton 2009; Wu et al. 2011a; Hyde et al. 2013). Ashton et al. (2009) estimated that there were 54 genera and 278 species in the family. In a subsequent series of papers, Wu et al. (2010, 2011a, b, [Bibr B70][Bibr B71]) revised Microthyriaceae by examining the generic type species and restricted Microthyriaceae to the species with morphological characteristics similar to *Microthyrium* Desm. Based on morphological characteristics, 11 genera and about 230 species were listed in this family (Wijayawardene et al. 2014), but in a subsequent outline of Ascomycota, only nine genera were accepted (Wijayawardene et al. 2018a). Recent studies accepted 11 genera in this family (Hongsanan et al. 2020; Wijayawardene et al. 2020).

Microthyriaceae have been poorly studied and there are few DNA sequences in public databases for this group of fungi. In the expanded multigene phylogeny of the Dothideomycetes, Microthyriaceae was not included because of the paucity of DNA sequences (Schoch et al. 2006). In the class-wide phylogenetic assessment of Dothideomycetes, Schoch et al. (2009) included Microthyriaceae, based on *Microthyriummicroscopicum* Desm. (type species of Microthyriaceae). One major contributing reason for the absence of DNA sequences is that few living cultures are available. As a result, researchers might have assumed that many of these species were obligate parasites and could not be cultured (Wu et al. 2011a). Later, Hongsanan et al. (2014) isolated cultures of *Chaetothyriotheciumelegans* Hongsanan & K.D. Hyde and *Tumidisporashoreae* Hongsanan & K.D. Hyde (Ariyawansa et al. 2015), but failed to observe anamorphs of the two species. Wu et al. (2014) tried to isolate fresh cultures of *Microthyriumpropagulensis* H.X. Wu & K.D. Hyde, but did not observe the germination of ascospores. Based on these situations, asexual genera of Microthyriaceae were recorded only from the literature. Before Wu revised Microthyriaceae, *Asterostomula* Theiss. and seven other genera were described as asexual morphs (Hyde et al. 2011; Wijayawardene et al. 2012). With the exclusion of many genera from Microthyriaceae (Wu et al. 2010, 2011a, b, c), only *Hansfordiella* S. Hughes was retained as an asexual genus in Microthyriaceae (Wijayawardene et al. 2018a), but this connection was not confirmed by molecular data because sequences of *Hansfordiella* were unavailable. Moreover, *Hansfordiella* was recorded as the asexual state of *Trichothyrium* Speg., which belongs to Trichothyriaceae (Ashton 2009; Hyde et al. 2011, 2013; Wijayawardene et al. 2012, 2017).

In the early 1990s, molecular methods, in particular DNA sequence data, provided opportunities for phylogenetic inference and have made a significant impact on the taxonomy and classification of fungi (Shenoy et al. 2007). More importantly, sequence analysis can potentially place an asexual-state taxon within an order or even link it with a teleomorph genus without having to observe the latter (e.g. in Berbee and Taylor 2001). The linkages between asexual and sexual genera have accumulated during implementation of the “One fungus: One name” concept, allowing the asexual genera to be placed in a natural biological framework of fungi (Wijayawardene et al. 2014, 2018a; Maharachchikumbura et al. 2015). However, the phylogenetic position of about 1530 genera in Ascomycota still remains incertae sedis (Wijayawardene et al. 2018a).

Aquatic hyphomycetes colonise allochthonous organic matter in fresh waters and are closely involved in the decomposition and conversion of biopolymers in aquatic habitats (Brlocher 1992). They are a polyphyletic group of fungi, mainly consisting of asexual morphs of Ascomycota and Basidiomycota, which have been identified, based on conidium morphology and conidiogenesis (Belliveau and Barlocher 2005). Molecular approaches applied to phylogeny of aquatic hyphomycetes place some genera in a defined class and found multiple origins of aquatic hyphomycetes. Specifically, seven strains (five species) of *Tetracladium* De Wild. showed close relationships to the Ascomycete orders Onygenales, Erysiphales and Leotiales (Nikolcheva 2002), but subsequently, Baschien and Szewzyk (2006) found *Tetracladium* located in Leotiomycetes, based on combined ITS and 28S analyses. Besides, studies of 31 species of aquatic hyphomycetes placed the majority (74%) within the Leotiomycetes (Belliveau and Barlocher 2005; Campbell et al. 2006). Duarte et al. (2015) constructed an ITS phylogenetic tree for 79 aquatic hyphomycetes, and found *Tricladium* Ingold and *Triscelophorus* Ingold are not monophyletic. Of course, with the availability of more and more reference sequences and the establishment of backbone trees of some classes, new aquatic hyphomycetes related to monophyly have been published with confirmed phylogenetic positions (Pratibha et al. 2015; Liu et al. 2016; Su et al. 2016; Qiao et al. 2018a; Wijayawardene et al. 2018a). Although these studies promoted phylogenetic development of aquatic hyphomycetes, the phylogenetic positions of most aquatic hyphomycetes have not been determined at the family level (Wijayawardene et al. 2018a).

In recent years, we have investigated the diversity and phylogeny of aquatic hyphomycetes from southern China which is a hot spot of world biodiversity, such as Yunnan, Sichuan, Guizhou, Guangdong and Hainan Provinces. Many new species collected from these regions have been described (Yang et al. 2011, 2012; Bai et al. 2013; Li et al. 2013, 2014; Guo et al. 2015, 2019; Qiao et al. 2017a, b, 2018b, 2019a, b, c, 2020; Peng et al. 2016; Yu et al. 2019; Zheng et al. 2020a, 2021a). In addition, several interesting isolates were collected. These isolates were cultured and sequenced and a BLAST search of its LSU sequences against data in GenBank revealed that the closest related taxa are in the genus *Microthyrium*. Based on the phylogenetic analysis combined with the internal transcribed spacers (ITS) and the large subunit (LSU) gene sequences and morphological features, two new genera and six new species are proposed within Microthyriaceae. In addition, we also collected *Isthmolongisporaquadricellularis* isolates and describe and illustrate it here.

## Methods

### Collection of samples, fungal isolation and morphological characterisation

Submerged leaves were collected from streams in Guangdong, Hainan Provinces and Tibet region. Samples were preserved in zip-locked plastic bags, labelled and transported to the laboratory at 4 °C. Each leaf was cut into several 3–4 × 4–5 cm-sized fragments, then these fragments were incubated on corn meal agar (CMA; 20 g cornmeal, 18 g agar, 40 mg streptomycin, 30 mg ampicillin, 1 litre distilled water) plates for 5 days at room temperature. Individual conidia were isolated using a sterilised toothpick under a BX51 microscope and cultivated on CMA plates. Morphological characteristics were observed from cultures growing on CMA and potato dextrose agar plates (PDA; 200 g potato, 20 g dextrose, 18 g agar, 1 litre distilled water) after incubation at 25 °C for one week. Microscopic photographs coming from CMA medium were taken with an Olympus BX51 microscope connected to a DP controller digital camera.

The pure cultures and dried cultures were deposited in the Herbarium of the Laboratory for Conservation and Utilization of Bio-Resources, Yunnan University, Kunming (YMF) and the China General Microbiological Culture Collection Center (CGMCC).

### DNA extraction, PCR amplification and sequencing

Genomic DNA was extracted from fresh mycelia grown on PDA at 25 °C as described by Turner et al. (1997). Fragments of the internal transcribed spacers (ITS) and the large subunit nuclear ribosomal RNA gene (LSU rRNA) were amplified with the following primer pairs: ITS4 and ITS5 for ITS (White et al. 1990) and LROR/LR7 (Vilgalys and Hester 1990), respectively. Each 25 μl PCR reaction volume consisted of 12.5 μl T5 Super PCR Mix (Beijing TsingKe Biotech Co., Ltd., Beijing, China), 1 μl of forward primer (10 µM), 1 μl of reverse primer (10 µM), 1μl DNA template, 5 μl of PCR buffer and 4.5 μl sterile water. The PCR thermal cycle programmes for the amplifications of these three DNA fragments followed those described in Su et al. (2016). PCR products were visualised on 1% agarose gel stained with Goldview (Geneshun Biotech, China) with D2000 DNA ladder (Realtimes Biotech, Beijing, China) and were then purified using a commercial Kit (Bioteke Biotechnology Co., Ltd., Beijing, China). DNA forward and reverse sequencing was performed with a LI-COR 4000L automatic sequencer with the same primers, using a Thermo Sequenase-kit as described by Kindermann et al. (1998). Finally, these new obtained sequences were deposited in the GenBank database at the National Center for Bio-technology Information (NCBI) and the accession numbers are listed in Table 1.

### Sequence alignment and phylogenetic analysis

Preliminary searches with newly-generated LSU and ITS gene sequences of these isolates against National Center for Biotechnology Information (NCBI) by the Basic Local Alignment Search Tool (BLAST) determined species closely related to our isolates. Based on this information, sequences of ITS and LSU were downloaded from Microthyriaceae and four sister orders belonging to Dothideomycetes, including 48 strains representing 35 species (Table 1), according to recent studies (Hongsanan et al. 2020; Iturrieta-González et al. 2020). *Schismatommadecolorans* (Erichsen) Clauzade & Vězda was used as the outgroup taxon.

**Table 1.** Species, strains and their corresponding GenBank accession numbers of sequences used for phylogenetic analyses. Newly-generated sequences are in bold.

Name	Strain	GenBank accession number	
		LSU	ITS
*Antidactylariaampulliforma*	CBS223.59	MH869386	MH857845
*Antidactylariaampulliforma*	P004	EU107302	—
*Antidactylariaampulliforma*	P038	EU107303	—
***Antidactylariaminifimbriata***	CGMCC 3.18825 = YMF 1.04578	**MK577808**	**MK569506**
*Chaetothyriotheciumelegans*	CPC 21375	KF268420	—
*Hamatisporaphuquocensis*	VICCF 1219	LC064073	LC064074
*Heliocephalaelegans*	MUCL 39003	HQ333478	HQ333478
*Heliocephalagracilis*	MUCL 41200	HQ333479	HQ333479
*Heliocephalanatarajanii*	MUCL 43745	HQ333480	HQ333480
*Heliocephalazimbabweensis*	MUCL 40019	HQ333481	HQ333481
***Isthmomycesdissimilis***	CGMCC 3.18826 = YMF 1.04604	**MK577811**	**MF740794**
***Isthmomyceslanceatus***	CBS 622.66	MH870563	MH858897
***Isthmomyceslanceatus***	YMF 1.04514	**MK577813**	**MK577895**
***Isthmomyceslanceatus***	CGMCC 3.18827	**MK577814**	**MK577896**
***Isthmomycesmacrosporus***	YMF 1.04518 = CGMCC 3.18824 = YMF 1.04794	**MK577812**	**MF740796**
***Isthmomycesoxysporus***	CGMCC 3.18821 = YMF 1.04513	**MK577810**	**MF740793**
*Lichenopeltellapinophylla*	CBS 143816	MG844152	—
*Microthyriumbuxicola*	MFLUCC 15-0212	KT306551	—
*Microthyriumbuxicola*	MFLUCC 15-0213	KT306552	—
*Microthyriumchinense*	HKAS 92487	KY911453	—
*Microthyriumfici-septicae*	NCYUCC 19-0038	MW063251	—
*Microthyriumfici-septicae*	MFLUCC 20-0174	MW063252	—
*Microthyriumilicinum*	CBS 143808	MG844151	—
*Microthyriummacrosporum*	CBS 143810	MG844159	—
*Microthyriummicroscopicum*	CBS 115976	GU301846	—
*Microthyriumpropagulensis*	IFRD 9037	KU948989	—
*Natipusilladecorospora*	AF236-1	HM196369	—
*Natipusillanaponense*	AF217-1	HM196371	—
*Neoanungiteaeucalypti*	CBS 143173	MG386031	MG386031
*Neoscolecobasidiumagapanthi*	CPC 28778	KY173517	KY173426
*Ochroconisdracaenae*	CPC 26115	KX228334	KX228283
*Parazalerionindica*	CBS 125443	MH874977	MH863483
*Phaeotrichumbenjaminii*	CBS 541.72	AY004340	MH860561
*Pseudomicrothyriumthailandicum*	MFLU 14-0286	MT741680	—
*Pseudopenidiellagallaica*	CBS 121796	LT984843	LT984842
*Pseudopenidiellapiceae*	CBS 131453	JX069852	JX069868
*Schismatommadecolorans*	DUKE 47570	AY548815	AY548808
*Scolecobasidiumtropicale*	CBS 380.87	KF156102	—
*Sympoventuriacapensis*	CBS 120136	KF156104	DQ885906
*Trichodelitschiabisporula*	CBS 262.69	GU348996	MH859305
***Triscelophorusanisopteriodeus***	CGMCC 3.18978 = YMF 1.04267	**MK577818**	**MK569511**
*Triscelophorusmonosporus*	CBS 440.54	MH868925	—
***Triscelophorussinensis***	YMF 1.04065	**MK577820**	**MK569513**
*Tumidisporashoreae*	MFLUCC 12-0409	KT314073	—
*Tumidisporashoreae*	MFLUCC 14-0574	KT314074	—
*Venturiainaequalis*	CBS 594.70	GU301879	KF156040
*Zeloasperisporiumficusicola*	MFLUCC 15-0221	KT387733	—
*Zeloasperisporiumhyphopodioides*	CBS 218.95	EU035442	EU035442
*Zeloasperisporiumsiamense*	IFRDCC 2194	JQ036228	—

For Microthyriaceae, the phylogenetic analysis was based on the combined ITS and LSU sequences. DNA sequence data of ITS and LSU were aligned using Clustal X 1.83 (Thompson et al. 1997) with the default parameters, then the consensus sequences were manually adjusted and linked through BioEdit v.7.0 (Hall 1999). Manual gap adjustments were carried out to improve the alignment and ambiguously-aligned regions were also excluded. We finally obtained the combined sequence matrix (Fasta file) generated by BioEdit v.7.0, containing 1119 nucleotide positions from two genes and the matrix was uploaded to TreeBASE (www.treebase.org; accession number: S28086). Bayesian Inference (BI) and Maximum Likelihood (ML) were used in this study for phylogenetic analyses. BI analysis was conducted with MrBayes v.3.2.2 (Ronquist et al. 2012) with NEXUS files converted by MEGA6 (Tamura et al. 2013). The Akaike Information Criterion (AIC) implemented in jModelTest 2.0 (Posada 2008) was used to select the best fit models after likelihood score calculations were done. GTR+F+I+G4 was estimated as the best-fit model under the output strategy of AIC. The parameters used were two simultaneous runs of 1,000,000 generations, four Markov chains, sampled every 500 generations. The 50% majority-rule consensus tree and posterior probability values (PP) were calculated after discarding the first 25% of the samples. ML analysis was computed by RAxML (Stamatakis 2006), using the GTR-GAMMA model. Maximum Likelihood bootstrap proportions (MLBP) were computed with 1000 replicates. Trees were visualised in FigTree 1.4.3 (http://tree.bio.ed.ac.uk/software/Figtree/, June 2021). Bayesian Inference posterior probabilities (BIPP) ≥ 0.9 and Maximum Likelihood bootstrap proportions (MLBP) ≥ 70% are indicated at nodes.

## Results

### Phylogenetic analyses

**Figure 1.**
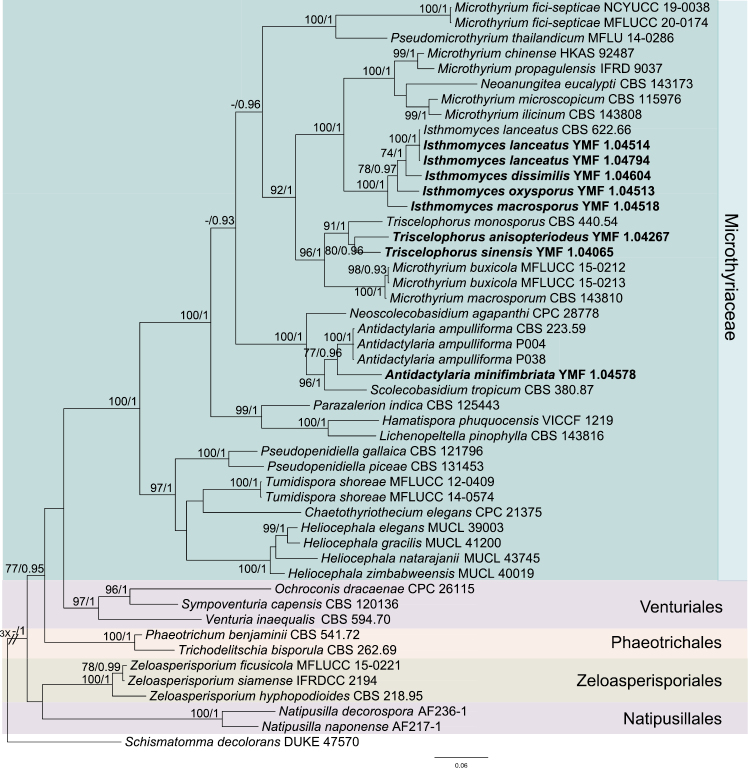
Phylogenetic tree generated by the Maximum Likelihood (ML) analysis using combined sequences of the nuclear large subunit (LSU) and the internal transcribed spacers (ITS) gene. Bootstrap support values for ML over 70% and Bayesian posterior probabilities greater than 0.9 are indicated above or below the nodes as MLBP/BIPP. *Schismatommadecolorans* strain DUKE 47570 is used as the outgroup. Novel species are indicated in bold.

The phylogenic tree, based on a combined sequence of the LSU and ITS, indicated that eight isolates belong to the Microthyriaceae (Fig. 1). After detailed observations of morphological features, these isolates were considered as six new species and one known species. In this tree, five isolates grouped with *Isthmolongisporalanceata* CBS 622.66 with good support (MLBP/BIPP = 100%/1.0). Combined with morphological differences, we proposed the new genus *Isthmomyces* to accommodate the three new species, designated as *I.dissimilis*, *I.macrosporus* and *I.oxysporus* and a new combination *I.lanceatus*. Two isolates, which clustered with *Triscelophorusmonosporus* CBS 440.54 (MLBP/BIPP = 91%/1.0), were considered as two new *Triscelophorus* species, designated as *Triscelophorusanisopteriodeus* and *T.sinensis*. The isolate YMF 1.04578 is phylogenetically close to *Isthmolongisporaampulliformis* (MLBP/BIPP = 77%/0.96). Considering morphological characters, we proposed a new genus *Antidactylaria* to accommodate the new species *A.minifimbriata* and the new combination *A.ampulliforma*.

### Taxonomy

#### Microthyriaceae

Taxon classificationFungiMicrothyrialesMicrothyriaceae

Sacc., Syll. fung. (Abellini) 2: 658 (1883).

09DDB1C1-BF0D-53FE-8EEA-C839B88DCE7B

81008

##### Description.

Hyde et al. 2013.

##### Type genus.

*Microthyrium* Desm., Annls Sci. Nat., Bot., sér. 2 15: 137 (1841).

##### Notes.

Microthyriales only contains a single family Microthyriaceae, based on morphology and phylogeny. Currently, eleven genera are accepted in Microthyriaceae, including three asexual genera (Hongsanan et al. 2020; Wijayawardene et al. 2020). The asexual morph of this family is characterised by having micronematous or macronematous, unbranched or branched, septate conidiophores, mono- to polyblastic, determinate or sympodial, clavate, subcylindrical, ampulliform or ovoid conidiogenous cells and solitary or in branched chains, acrogenous or acropleurogenous, aseptate to multi-septate conidia. In this study, we erected two new asexual genera, *Antidactylaria* and *Isthmomyces* and recognised six new asexual species in Microthyriaceae, based on DNA sequences at two gene fragments. In addition, two new combinations are proposed in Microthyriaceae combined morphology and phylogeny.

#### Antidactylaria

Taxon classificationFungiMicrothyrialesMicrothyriaceae

Z.F. Yu, M. Qiao & R.F. Castañeda gen. nov.

EC48F58B-DA15-5399-A3ED-D3A3046E59B3

Index Fungorum number: IF555876

Facesoffungi Number No: FoF05734

##### Etymology.

Greek, *Anti*, meaning against, Latin, *dactylaria*, referring to the genus *Dactylaria*.

##### Description.

Asexual morph hyphomycetous. *Mycelium* superficial and immersed. *Conidiophores* macronematous, erect, unbranched, septate, hyaline, sometimes reduced to conidiogenous cells. *Conidiogenous cells* denticulate, polyblastic, sympodial elongated, integrated, terminal determinate or indeterminate, hyaline. Conidial secession rhexolytic. *Conidia* solitary, acrogenous, narrow obclavate, cylindrical to fusiform, navicular, attenuate towards the apex, rostrate, unicellular or septate, hyaline or subhyaline, smooth-walled, with a minute basal frill. Sexual state: unknown.

##### Type species.

*Antidactylariaminifimbriata* Z.F. Yu, M. Qiao & R.F. Castañeda.

##### Notes.

*Antidactylaria* is superficially similar to the genus *Dactylaria* Sacc. in morphology. The genus *Dactylaria*, typified with *D.purpurella* (Sacc.) Sacc., is characterised by unbranched, septate, hyaline or pigmented conidiophores, denticulate, integrated, mostly terminal, sympodially extending conidiogenous cells and cylindrical, fusiform, filiform, ellipsoid, clavate, obclavate, unicellular or septate, hyaline or pale pigmented conidia that are liberated with schizolytic secession (Goh and Hyde 1997; Paulus et al. 2003; Seifert et al. 2011). However, the rhexolytic conidial secession, observed in *Antidactylaria*, is absent in *Dactylaria*. Paulus et al. (2003) discussed the conidiogenous event as an important criterion for generic delimitation. In addition, phylogeny analysis showed that *Antidactylaria* species belong to Microthyriales, while *Dactylaria* species belong to Helotiales.

#### Antidactylaria ampulliforma

Taxon classificationFungiMicrothyrialesMicrothyriaceae

(de Hoog & Hennebert) Z.F. Yu, M. Qiao & R.F. Castañeda comb. nov.

DB0B84CC-A39C-5614-9C32-992D1BCF8999

108094

Isthmolongispora ampulliformis (Tubaki) de Hoog & Hennebert, Proc. K. Ned. Akad. Wet., Ser. C, Biol. Med. Sci. 86(3): 346 (1983)
Diplorhinotrichum ampulliforme Tubaki, J. Hattori bot. Lab. 20: 159 (1958)

##### Description.

Matsush. 1975

##### Notes.

*Antidactylariaampulliforma* was originally isolated by Tubaki from leaves of *Cocosnucifera* and was described as *Diplorhinotrichum* species (Tubaki 1958). In 1983, de Hoog and Hennebert included it in the genus *Isthmolongispora* after examining its morphological character. In this study, *A.ampulliforma* is phylogenetically close to *A.minifimbriata* and they are very similar in morphology. Therefore, we assigned it in the newly-established genus *Antidactylaria* as a new combination.

#### Antidactylaria minifimbriata

Taxon classificationFungiMicrothyrialesMicrothyriaceae

Z.F. Yu, M. Qiao & R.F. Castañeda sp. nov.

322B49F0-8D58-5A7B-9822-F4387DBEAFE1

Index Fungorum number: IF556121

Facesoffungi Number No: FoF05735

Figs 2 , 9a

##### Etymology.

Latin, *mini*, meaning very small, minute, *fimbriata*, referring to edged, delicately toothed, fringe or frill that remained on the conidial base after rhexolytic secession.

**Figure 2.**
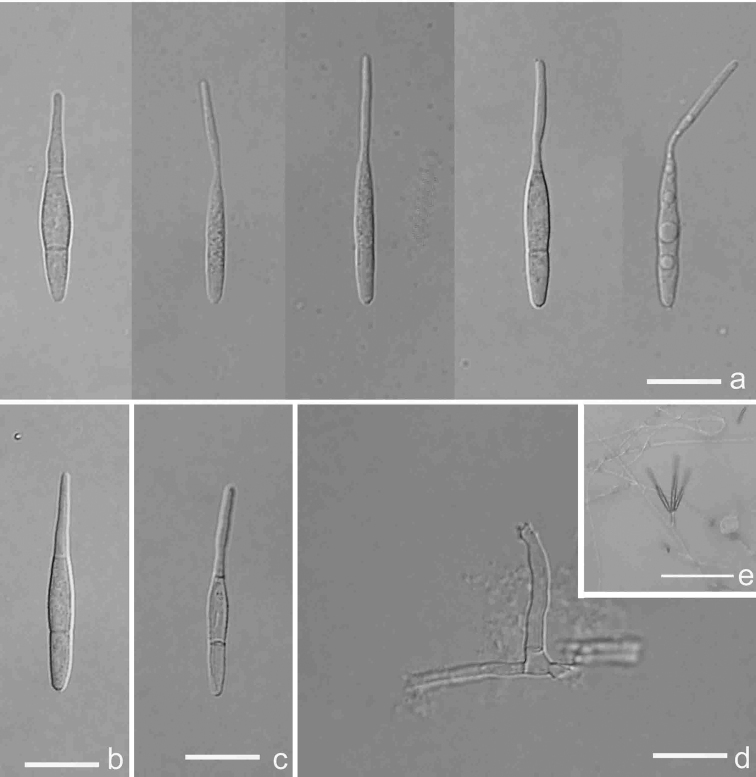
*Antidactylariaminifimbriata* (Holotype YMF 1.04578) **a–c** conidia **d** conidiophore and conidiogenous cell **e** conidia on conidiophore under low objective. Scale bars: 10 µm (**a–d**); 50 µm (**e**).

##### Description.

Asexual morph hyphomycetous. *Colonies* on CMA white to rosy buff, reverse buff, attaining 2.7 cm diam. after 20 days at 25 °C. *Mycelium* partly superficial, partly immersed, composed of branched, slender, septate, hyaline, smooth-walled hyphae. *Conidiophores* semi-macronematous, mononematous, cylindrical, straight or slightly flexuous, unbranched, 0–1(–2)-septate, hyaline or pale brown, smooth, sometimes reduced to conidiogenous cells. *Conidiogenous cells* polyblastic, sympodial elongated, terminal, denticulate, denticles cylindrical, minute fringed. *Conidia* solitary, acrogenous, narrow obclavate, cylindrical to fusiform, attenuate, rostrate or caudate towards the apex, 27.7–40 × 2.5–3.3 µm, rostrum 10–19 × 1–1.8 µm, 2-septate, hyaline to subhyaline, smooth-walled, with a minute basal frill. Sexual state: unknown.

##### Type.

**China**, Hainan Province, Diaoluoshan National Forest Park, on submerged leaves, April 2014, Z.F Yu. Holotype YMF 1.04578, preserved in a metabolically-inactive state (deep freezing) in the Conservation and Utilization of Bio-Resources in Yunnan. Ex-type culture CGMCC 3.18825.

##### Notes.

Morphologically, *Antidactylariaminifimbriata* is similar to *A.ampulliforma* (= *Isthmolongisporaampulliformis*) in conidial shape, but can be easily distinguished from it by having wider conidia (2.5–3.3 vs. 2.0–2.5 µm) and longer rostrum (10.0–19.0 vs. 6.0–10.0 µm) (Yen et al. 2017).

#### Isthmomyces

Taxon classificationFungiMicrothyrialesMicrothyriaceae

Z. F. Yu, M. Qiao & R. F. Castañeda gen. nov.

21A5D891-7983-52C2-AFCE-3D3B854E8E48

Index Fungorum number: IF556126

Facesoffungi Number No: FoF05740

##### Etymology.

Latin, *isthmus*, Greek (isthmós, “neck”) meaning a narrow cellular structure that connects two larger bodies or cells, Greek, *myces*, referring to fungus.

##### Description.

Asexual morph hyphomycetous. *Mycelium* superficial and immersed. *Conidiophores* macronematous, mononematous, erect, unbranched, smooth, pale brown or hyaline, septate, sometimes reduced to conidiogenous cells. *Conidiogenous cells* polyblastic, denticulate, integrated, terminal, sympodial extended. *Conidial secession* schizolytic. *Conidia* acrogenous, isthmosporous, composed two cellular isthmic-segment obclavate, clavate, pyriform, obpyriform, lageniform, subulate fusiform to navicular to lanceolate, unicellular or septate, smooth, hyaline, connected by a very narrow, distinct or inconspicuous isthmus. Sexual state: unknown.

##### Type species.

*Isthmomycesoxysporus* Z.F. Yu, M. Qiao & R.F. Castañeda.

##### Notes.

*Isthmomyces* is similar to the genus *Isthmolongispora* Matsush. in morphology. *Isthmolongispora* was established with *I.intermedia* Matsush. as type species (Matsushima 1971). The genus is characterised by denticulate, sympodially-extending conidiogenous cells and isthmospore conidia made of two or several cellular structures, which are connected by very narrow isthmuses. In this study, specimens with two and more cellular isthmic-segments were collected, respectively. Phylogenetic analysis inferred from two loci showed that our isolates grouped together with *Isthmomyceslanceatus* (*Isthmolongisporalanceata*) in Microthyriaceae. Combining morphological character and phylogenetic analysis, we finally erected the new genus *Isthmomyces* to accommodate these isolates and *I.lanceata*.

#### Isthmomyces dissimilis

Taxon classificationFungiMicrothyrialesMicrothyriaceae

Z. F. Yu, M. Qiao & R. F. Castañeda sp. nov.

24450751-05DE-5901-BACB-8556C8AE4369

Index Fungorum number: IF556129

Facesoffungi Number No: FoF05743

Figs 3 , 9b

##### Etymology.

Latin, *dissimilis*, referring to the variation of the conidial shape related to the generic concept of the genus.

##### Description.

Asexual morph hyphomycetous. *Colonies* on CMA white to dark salmon, reverse pale yellow, attaining 2.5 cm diam. after 20 days at 25 °C. *Mycelium* superficial or immersed, composed of branched, septate, brown, hyphae. *Conidiophores* macronematous, mononematous, erect, straight, unbranched or slightly branched, 0–1- septate, smooth, subhyaline13.8–51 × 2.3–3.2 µm. *Conidiogenous cells* polyblastic, ampulliform to cylindrical, sympodial extended, integrated, terminal, subhyaline. *Conidia* acrogenous, isthmospore, with inconspicuous isthmus, (isthmus mostly reduced to being constricted at the septa) subhyaline, guttulate, smooth, composed of 2–3-cellular isthmic-segments, more or less symmetrical: A) the larger isthmospore with 2-cellular isthmic-segments: i) basal isthmic-segment cylindrical-fusiform, truncate below, 1–3 septate, 35–60 × 4–4.5 µm, ii) apical isthmic-segment fusiform, rounded at the tip, 0–2 septate, 17–36.5 × 4–4.5 µm; total long 70–95 µm. B) the smaller isthmospore with 2-cellular isthmic-segments: i) basal isthmic-segment cylindrical-fusiform, truncate below, 0–1 septate, 23–33 × 3.5–4.5 µm; ii) apical isthmic-segment fusiform, rounded at the tip, 0–1 septate, 17–22 × 3.5–4.5 µm; total long 47–57 µm. C) isthmospore with 3-cellular isthmic-segments: i) basal isthmic-segment fusiform, truncate below, 2–3-septate, 18.5–38.5 × 2.8–5.0 µm; ii) central isthmic-segment cylindrical-fusiform, 2–3-septate, 20.1–44.5 × 3.0–6.2 µm; iii) apical isthmic-segment fusiform, rounded or obtuse at the tip, 0–2-septate, 17.4–31.6 × 2.3–4.8 µm. Sexual state: unknown.

**Figure 3.**
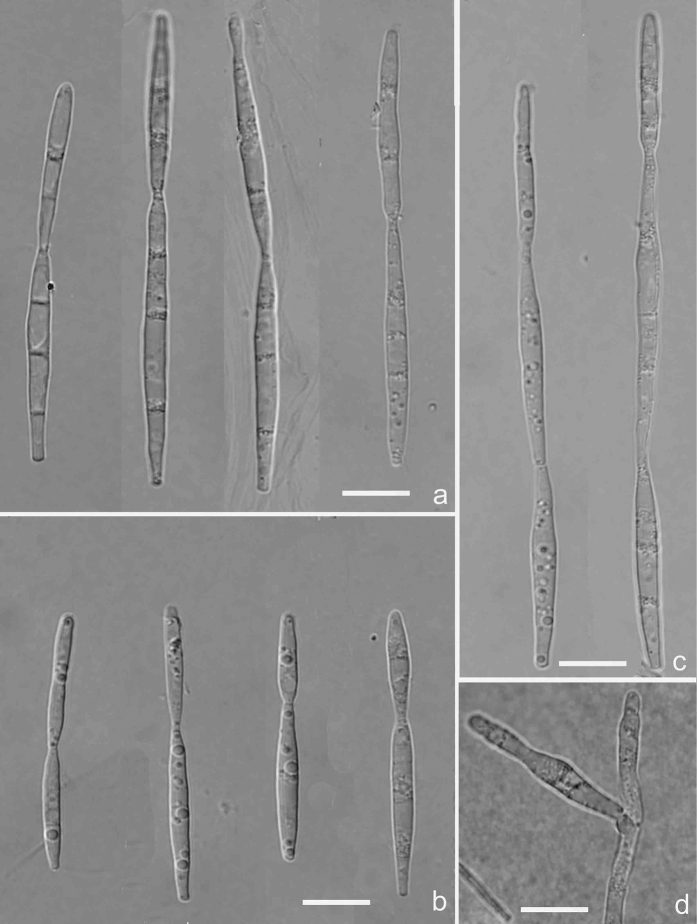
*Isthmomycesdissimilis* (Holotype YMF 1.04604) **a** the larger isthmospore with 2-cellular isthmic-segments **b** the smaller isthmospore with 2-cellular isthmic-segments **c** isthmospores with 3-cellular isthmic-segments **d** conidiogenous cell and developing conidia. Scale bars: 10 µm (**a–d**).

##### Type.

**China**, Hainan Province, Diaoluo Mountain Nature Reserve, on submerged leaves, August 2015, J. Peng. Holotype YMF 1.04604, preserved in a metabolically-inactive state (deep freezing) in the Conservation and Utilization of Bio-Resources in Yunnan. Ex-type culture CGMCC 3.18826.

##### Notes.

The new species, *Isthmomycesdissimilis*, varies in conidial shape. Although it has 3-cellular isthmic-segment conidia, its isthmic-segment is not as distinct as *Isthmolongispora* species. However, the cells of *Isthmolongispora* are bead-like, while those of *I.dissimilis* are cylindrical to fusiform.

#### Isthmomyces lanceatus

Taxon classificationFungiMicrothyrialesMicrothyriaceae

(de Hoog & Hennebert) Z. F. Yu & R. F. Castañeda comb. nov.

EAB2CAFF-07C7-5BFB-8181-FF31526587F4

Index Fungorum number: IF556158

Facesoffungi Number No: FoF05757

Figs 4 , 9c

Isthmolongispora lanceata de Hoog & Hennebert, Proc. K. Ned. Akad.Wet., Ser. C, Biol. Med. Sci. 86(3): 343 (1983).

##### Description.

Asexual morph hyphomycetous. *Colonies* on CMA white to dark salmon, reverse pale brown, attaining about 2 cm diam. after 20 days at 25 °C. *Mycelium* partly superficial, partly immersed, composed of branched, septate, slender, hyaline hyphae. *Conidiophores* macronematous, mononematous, cylindrical, erect, straight, unbranched, 0–1- septate, smooth, hyaline, up to 30 µm long, 3–3.5 µm wide. *Conidiogenous cells* polyblastic, cylindrical, denticulate, sympodial extended, integrated, terminal, hyaline. *Blastoconidia* isthmospore, somewhat fusiform, hyaline or subhyaline, smooth, thin-walled, 21.3–39.7 µm long, strongly constricted at the median septum, narrow, tiny, made of two cellular isthmic-segments: i) basal isthmic-segment narrow-clavate, sometimes cylindrical-clavate, truncated at the base, unicellular, 0–1-septate, 12.5–18.5 × 3.0–4.8 µm; ii) apical isthmic-segment broadly obclavate, obspathulate, rounded at the tip, unicellular, 0–1-septate, 13.0–30.0 × 2.3–3.8 µm. *Arthroconidia* often formed in the aerial mycelium, disarticulated from fertile hyphae. Sexual state: unknown.

**Figure 4.**
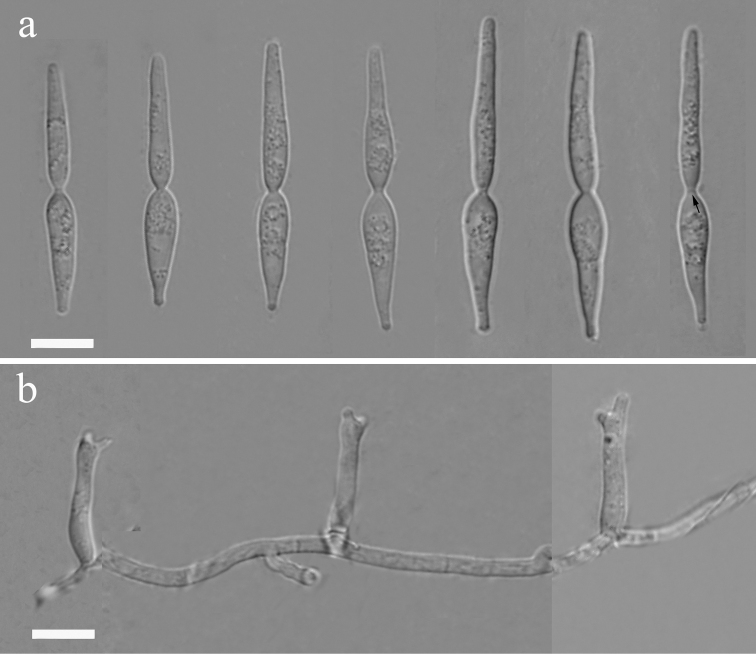
*Isthmomyceslanceatus* (YMF 1.04794) **a** conidia **b** conidiophores and conidiogenous cells. Scale bars: 10 µm (**a, b**). The arrow indicates septum inside isthmic-segments.

##### Type.

**China**, Tibet, Nanyigou Scenic Area, on submerged leaves, October 2016, Z.F. Yu, YMF 1.04794 = CGMCC 3.18827. **China**, Yunnan Province, Jade Dragon Snow Mountain, on submerged leaves, September 2015, J. Peng, YMF 1.04514.

##### Notes.

*Isthmomyceslanceatus* was first isolated by Beverwijk from leaf of *Castaneavesca* in steam (Hoog and Hennebert 1983). However, the taxonomic status of this species was Ascomycota*incertae sedis*. In this study, this is the first report of *I.lanceatus* isolated from Asia. Morphologically, the conidia of our isolates are larger than the holotype CBS 622.66. Our phylogenetic analysis of combined LSU and ITS sequences reveals that the phylogenetic position of *I.lanceatus* is in Microthyriaceae and *I.lanceatus* is close to *I.dissimilis* in this tree.

#### Isthmomyces macrosporus

Taxon classificationFungiMicrothyrialesMicrothyriaceae

Z. F. Yu, M. Qiao & R. F. Castañeda sp. nov.

062C80A1-0A8C-537C-A2DE-B8EC3CD4504F

Index Fungorum number: IF556128

Facesoffungi Number No: FoF05742

Figs 5 , 9d

##### Etymology.

Greek, *macrosporus*, referring to the large, great conidia.

##### Description.

Asexual morph hyphomycetous. *Colonies* on PDA amber to fawn, reverse fawn, attaining 2 cm diam. after 20 days at 25 °C. *Mycelium* mostly immersed, composed of branched, septate, slender, colourless hyphae. *Conidiophores* macronematous, mononematous, cylindrical, erect, straight, unbranched, 0–1-septate, smooth, pale brown, 25–35 × 3.0–3.5 µm. *Conidiogenous cells* polyblastic, cylindrical, denticulate, sympodial extended, integrated, terminal, pale brown or subhyaline. *Conidia* acrogenous, isthmospore, long fusiform, hyaline, smooth, 36.5–73.0 µm long, strongly constricted at the conspicuous, narrow, tiny central isthmus, sometime not differentiated, composed of two cellular isthmic-segments: i) basal isthmic-segment clavate, truncated at the base, 1-septate, hyaline or subhyaline, smooth, 19.2–31.1 × 4.5–6.7 µm; ii) apical isthmic-segment 0–1-septate, narrow obclavate, sometimes sub-obspathulate, rounded at the tip, unicellular, guttulate, hyaline or subhyaline, smooth, 21.1–42.0 × 3.3–5.4 µm. Sexual state: unknown.

**Figure 5.**
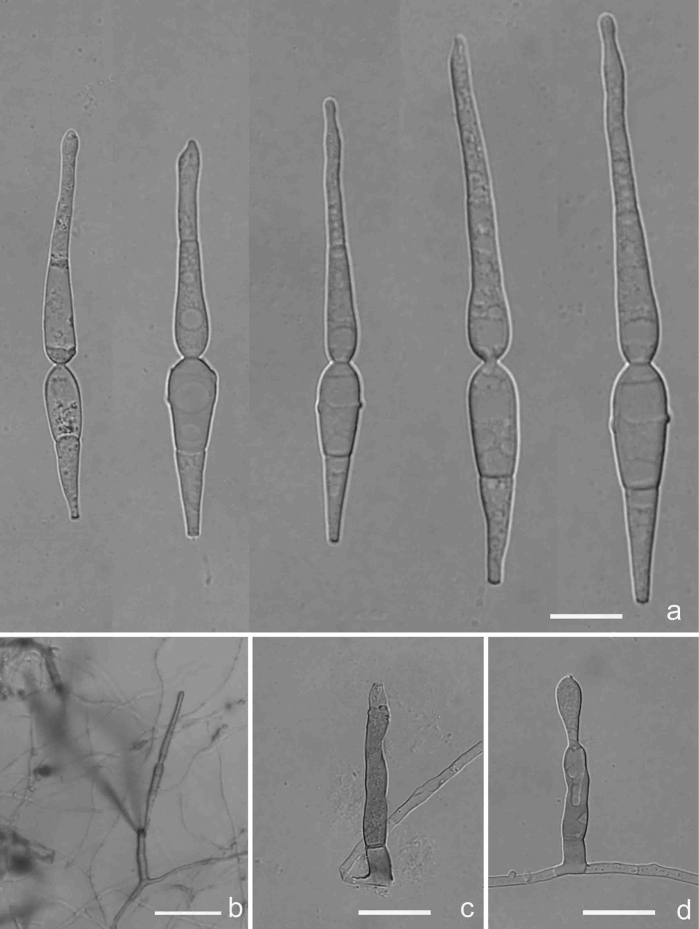
*Isthmomycesmacroporus* (Holotype YMF 1.04518) **a** conidia **b** conidiophore with conidia under low objective **c** conidiophore and conidiogenous cell **d** conidiophore and developing conidia. Scale bars: 10 µm (**a, c, d**); 50 µm (**b**).

##### Type.

**China**, Hainan Province, Limu Mountain National Conservation Area, on submerged leaves, April 2015, J. Peng. Holotype YMF 1.04518, preserved in a metabolically-inactive state (deep freezing) in the Conservation and Utilization of Bio-Resources in Yunnan. Ex-type culture CGMCC 3.18824.

##### Notes.

Phylogenetically, *Isthmomycesmacrosporus* is close to *I.dissimilis* and *I.lanceatus*. However, *I.macrosporus* is different from all species within this genus by having larger conidia, obviously brown conidiophores and few denticulate conidiogenous cells (Hoog and Hennebert 1983).

#### Isthmomyces oxysporus

Taxon classificationFungiMicrothyrialesMicrothyriaceae

Z. F. Yu, M. Qiao & R. F. Castañeda sp. nov.

9DB45750-0D9E-5C15-B817-F66FA71DC009

Index Fungorum number: IF556127

Facesoffungi Number No: FoF05741

Figs 6 , 9e

##### Etymology.

Greek, *oxys*, meaning sharp, keen, *sporum*, referring to the conidia.

##### Description.

Asexual morph hyphomycetous. *Colonies* on CMA pale mouse grey to dark mouse grey, reverse olivaceous-grey, attaining about 2 cm diam. after 20 days at 25 °C. *Mycelium* mostly immersed, composed of branched, septate, subhyaline to hyaline hyphae. *Conidiophores* macronematous, mononematous, cylindrical, erect, smooth, 0–1-septate, subhyaline to hyaline, mostly reduced to conidiogenous cells, up to 30 µm long, 2.5–3 µm wide, arising from the creeping hyphae. *Conidiogenous cells* polyblastic, cylindrical, denticulate, integrated, terminal, sympodial extended, hyaline. *Conidia* isthmospore, fusiform, hyaline, smooth, 20.5–25.5 µm long, strongly constricted at the narrow, tiny central isthmus, composed of two cellular isthmic-segments: i) basal isthmic-segment broadly clavate to clavate, unicellular, hyaline 9.7–13 × 2.0–4.0 µm; ii) apical isthmic-segment narrow obclavate to obclavate, obpyriform or rarely lecythiform, unicellular, hyaline, 9.0–13.0 × 2.0–3.0 µm. Sexual state: unknown.

**Figure 6.**
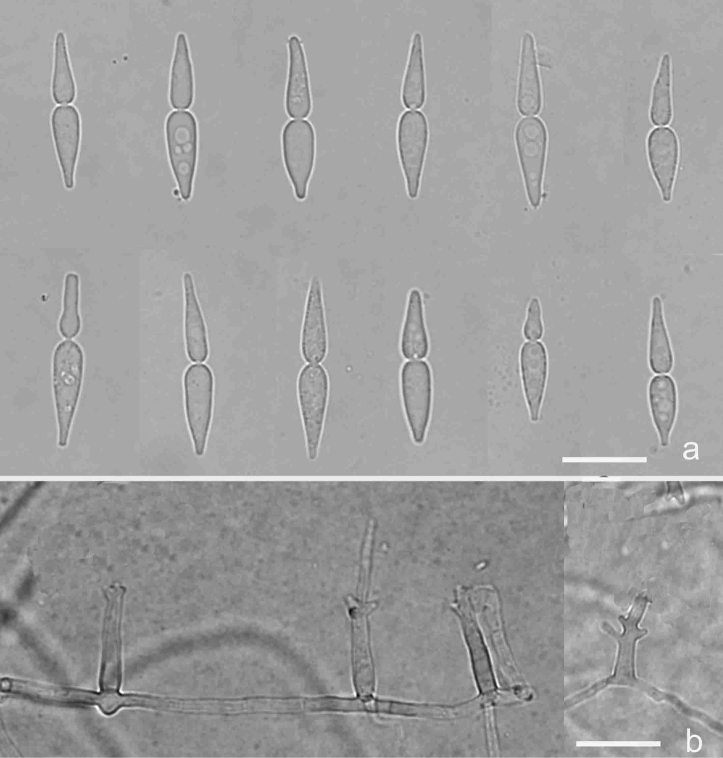
*Isthmomycesoxysporus* (Holotype YMF 1.04513) **a** conidia **b** conidiophores and conidiogenous cells. Scale bars: 10 µm (**a, b**).

##### Type.

**China**, Hainan Province, Diaoluo Mountain Natural Reserve, on submerged leaves, August 2015, J. Peng. Holotype YMF 1.04513, preserved in a metabolically-inactive state (deep freezing) in the Conservation and Utilization of Bio-Resources in Yunnan. Ex-type culture CGMCC 3.18821.

##### Notes.

Morphologically, *Isthmomycesoxysporus* resembles *Isthmolongisporaasymmetrica* Aramb. & Cabello in having both tapering isthmic-segment ends, but *Is.asymmetrica* has asymmetrical conidia, in which the basal isthmic-segment is longer (17–20 µm long) (Arambarri et al. 1987). Besides, *I.oxysporusis* is somewhat similar to *Is.rotundata* Matsush. in conidial sizes, but the apical isthmic-segments in *Is.rotundatus* are rounded at the tip (Matsushima 1987).

#### Triscelophorus

Taxon classificationFungiMicrothyrialesMicrothyriaceae

Ingold, Trans. Br. mycol. Soc. 26(3–4): 151 (1943).

1E61791C-5F24-529B-95CD-D664B202D90B

10320

##### Description.

Ingold 1943.

##### Type species.

*Triscelophorusmonosporus* Ingold, Trans. Br. mycol. Soc. 26(3–4): 152 (1943).

##### Notes.

*Triscelophorus* was established by Ingold, with *T.monosporus* as type species (Ingold 1943). The genus is characterised by macronematous, mononematous, erect, straight or flexuous, sometimes sinuate, septate, unbranched or sparingly branched, hyaline, smooth conidiophores. The conidiogenous cells are monoblastic, sometimes sympodially extended, integrated, hyaline that produce a solitary, acrogenous, septate, staurospore composed of a main axis and 3 or more branches verticillate arranged from the basal cell of the main axis (Ingold 1943; Seifert et al. 2011). Duarte et al. (2015) found that *Triscelophorus* was polyphyletic, based on ITS analysis, but our phylogenetic analysis, based on two-loci and ITS, showed the genus should be monophyletic. For more details, refer to Discussion.

#### Triscelophorus anisopteriodeus

Taxon classificationFungiMicrothyrialesMicrothyriaceae

Z. F. Yu, M. Qiao & R. F. Castañeda sp. nov.

D6DCEEDA-304D-5915-AA53-8FC26E1B53E8

Index Fungorum number: IF556148

Facesoffungi Number No: FoF05747

Figs 7 , 9f

##### Etymology.

Latin, *anisopteriodeus*, referring to the resemblance of the conidial body to an adult of *Anisoptera* sp.

##### Description.

Asexual morph hyphomycetous. *Colonies* on CMA, attaining about 1 cm diam. after 20 days at 25 °C, light smoky grey. Reverse smoky grey. *Mycelium* superficial and immersed, composed of branched, septate, hyaline hyphae. *Conidiophores* macronematous, mononematous, cylindrical, erect, flexuous, unbranched, smooth, hyaline, up to 20–110 µm long. *Conidiogenous cells* monoblastic, cylindrical, terminal, integrated, determinate, smooth, hyaline. *Conidia* solitary, acrogenous, staurospore, septate, composed of a main axis and 2–4 lateral branches: i) the main axis elongate obclavate, 2–4-septate, straight, smooth, hyaline, 31.2–48 × 3–5.2 µm; ii) 2–4-lateral branches obclavate to broad obclavate, straight, smooth, hyaline, all arising divergent, unequal, from the basal cell of the main axis: ii a) upper two lateral branches, 2–3-septate, 8.2–38.7 × 2.5–4.8 µm, more or less opposite, arranged just below the supra-basal septum; ii b) lower lateral branches, 0–1-septate, 14–20 × 5–5.5 µm, sequential opposite arranged near the middle of the basal cell. Sexual state: unknown.

**Figure 7.**
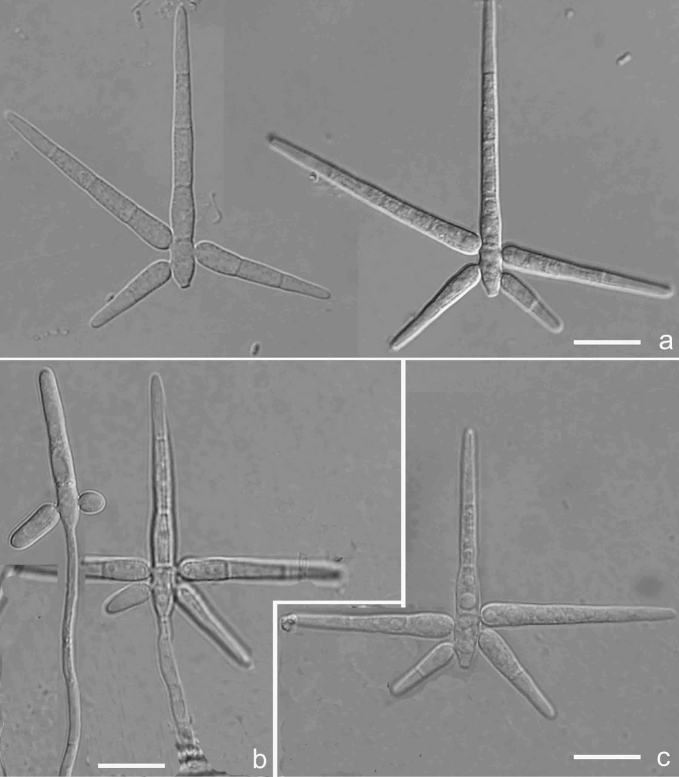
*Triscelophorusanisopteriodeus* (Holotype YMF 1.04267) **a, c** conidia **b** conidiophores with conidia. Scale bars: 10 µm (**a–c**).

##### Type.

**China**, Hainan Province, Limu Mountain Nature Reserve, on submerged leaves, April 2015, J. Peng. Holotype YMF 1.04267, preserved in a metabolically-inactive state (deep freezing) in the Conservation and Utilization of Bio-Resources in Yunnan. Ex-type culture CGMCC 3.18978.

##### Notes.

*Triscelophorusanisopteriodeus* is differentiated from other known *Triscelo2*like a dragonfly-shape (Seifert et al. 2011). Four lateral branches are not arising from the same level at the basal cell of main axis. Two shorter ones are lower and two longer ones are upper. Amongst conidia of *Triscelophorus* spp., three lateral branches are often growing in a whorl, while 2 lateral branches are in pairs. Four lateral branches in pairs in *T.anisopteriodeus* make it easily recognisable. Morphologically, *T.anisopteriodeus* is similar to *Triramulisporaduobinibrachiata* K. Ando in conidial shape, but *T.anisopteriodeus* has larger size of conidia (main axis: 31.2–48 × 3–5.2 vs. 19–36 × 2.5–3.5 µm) and more septa in branches (Ando 1993).

#### Triscelophorus sinensis

Taxon classificationFungiMicrothyrialesMicrothyriaceae

Z. F. Yu, M. Qiao & R. F. Castañeda sp. nov.

6E8DAD53-69CA-5DC2-8CAE-6064F32FB707

Index Fungorum number: IF558520

Figs 8 , 9g

##### Etymology.

Latin, *sinensis*, referring to the country of origin, China.

##### Description.

Asexual morph hyphomycetous. *Colonies* on CMA, attaining about 1 cm diam. after 20 days at 25 °C, pale mouse grey to dark mouse grey. *Mycelium* superficial and immersed, composed of branched, septate, hyaline hyphae. *Conidiophores* macronematous, mononematous, lateral or terminal, cylindrical, erect, flexuous, separate, smooth, hyaline, up to 12–38 µm long, 1.0–2.4 µm wide. *Conidiogenous cells* monoblastic, cylindrical, terminal, integrated, determinate, smooth, hyaline. *Conidia* solitary, acrogenous, staurospore, septate, composed of a main axis and 2–3 lateral branches: i) the main axis obclavate, 2(–3)-septate, slightly constricted at the septa, straight, smooth, hyaline, 17.5–30.0 × 3.5–5.0 µm; ii) 2–3-lateral branches obclavate, (0–)1-septate, slightly constricted at the septa, straight, smooth, hyaline, 8.5–21.0 × 3.0–4.5 µm, arising from the basal cell of the main axis arranged in a regular or irregular verticillate. Sexual state: unknown.

**Figure 8.**
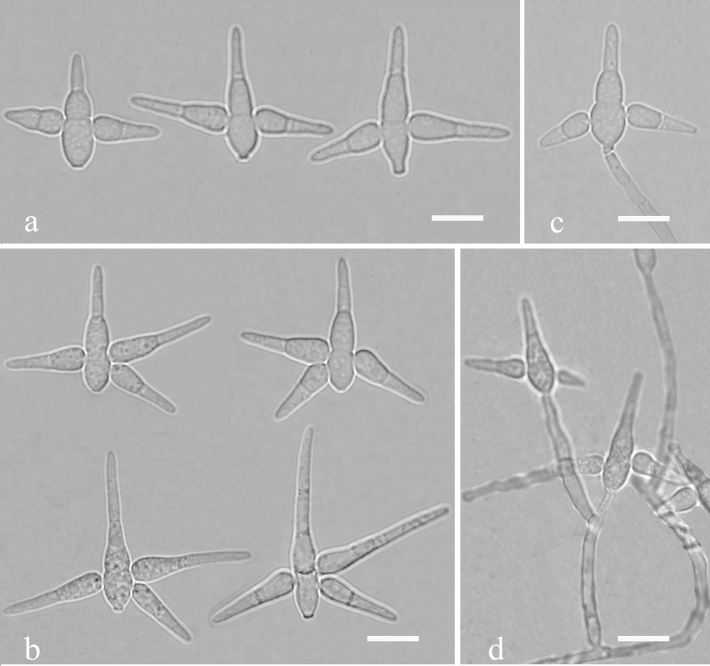
*Triscelophorussinensis* (Holotype YMF 1.04065) **a, b** conidia **c, d** conidiophores with conidia. Scale bars: 10 µm (**a–d**).

##### Type.

**China**, Guangdong Province, Guangzhou, on submerged leaves, September 2011, G.Z. Yang. Holotype YMF 1.04065, preserved in a metabolically-inactive state (deep freezing) in the Conservation and Utilization of Bio-Resources in Yunnan.

**Figure 9.**
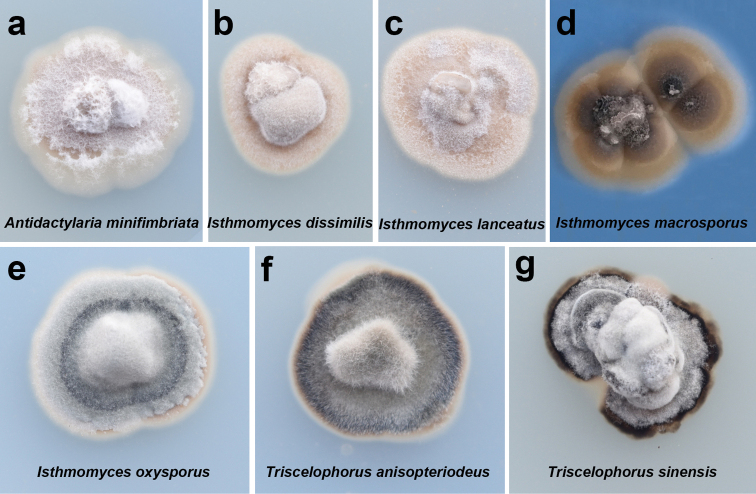
Cultural characters of all species in this study after 20 days on PDA at 25 °C.

##### Notes.

In morphology, *Triscelophorussinensis* is somewhat similar to *T.ponapensis* in conidia, both having 2–3 lateral arms (Matsushima 1981). However, *T.ponapensis* has shorter (main axis: 12–26 µm; lateral arms: 8–15 µm) and more septate (main axis: 2–4-septate; lateral arms: 1–4-septate) conidia.

#### Isthmolongispora quadricellularia

Taxon classificationFungiMicrothyrialesMicrothyriaceae

Matsush., Icon. microfung. Matsush. lect. (Kobe): 90 (1975).

D6FC3437-5F7A-5A7E-8C6F-B42C3E666957

315952

Fig. 10

##### Description.

Asexual morph hyphomycetous. *Colonies* on CMA white, gradually turning brown, reverse white to pale brown, attaining about 2.5 cm diam. after 20 days at 25 °C. *Mycelium* partly superficial, partly immersed, composed of branched, septate, slender, hyaline hyphae. *Conidiophores* macronematous, mononematous, cylindrical, erect, straight, unbranched, aseptate, smooth, hyaline, 3.9–9.0 × 2.0–3.2 µm. *Conidiogenous cells* short, terminal, cylindrical, denticulate, integrated, hyaline. *Conidia* solitary, smooth, beaded, tapering towards both ends, 4–7-celled, generally 5–6-celled, hyaline, 44–88 × 3.5–5.0 µm. Sexual state: unknown.

**Figure 10.**
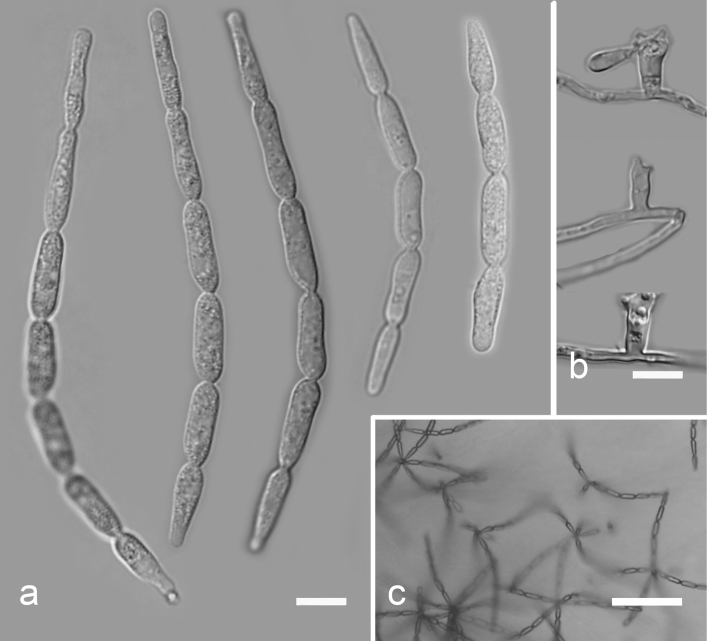
*Isthmolongisporaquadricellularia* (YMF 1.04794) **a** conidia **b** conidiophores and conidiogenous cells **c** conidia under low power microscopy. Scale bars: 10 µm (**a, b**); 50 µm (**c**).

##### Type.

**China**, Hainan Province, Jianfengling National Nature Reserve, on submerged leaves, Jun 2011, G.Z. Yang, YMF 1.04794, YMF 1.04011, YMF 1.04016, YMF 1.04019, preserved in a metabolically-inactive state (deep freezing) in the Conservation and Utilization of Bio-Resources in Yunnan.

##### Notes.

*Isthmolongisporaquadricellularia* was first described by Matsush. in 1975 from Japan. Subsequently, this species has been isolated from leaves many times in Taiwan. However, no sequences of *I.quadricellularia* are available in the public database. In this study, it is the first time that *I.quadricellularia* has been isolated from the aquatic environment. In addition, we also submitted sequence data for this species to the public database (SSU: MT507103–105; LSU: MT507107–110; ITS: OL412746–749).

## Discussion

China is considered an important reservoir of Asian biodiversity (Myers et al. 2000); it is estimated that this area harbours an inestimable diversity of fungi. In recent years, more and more new genera and species have been identified and classified for the application of phylogenetic analysis and have led to a significant expansion of species in Dothideomycetes (Zheng et al. 2019, 2020b, 2021b; Yang et al. 2021). However, comparatively speaking, aquatic hyphomycetes have been poorly investigated. In recent years, we have been investigating the diversity of aquatic hyphomycetes from southern China. During this process, several interesting isolates have been collected. After studying in detail, two new asexual genera and six new asexual species have been described in Microthyriaceae.

*Triscelophorus* Ingold was established with *T.monosporus* Ingold as type species; now, eight species have been accepted in this genus (Ingold 1943; Wijayawardene et al. 2017). However, the positions of *Triscelophorus* in ordinal and familial levels are still unclear. In this study, two isolates which have similarity to *Triscelophorus* species in morphology were collected. For further study, the two isolates were identified as two new species of *Triscelophorus*, named as *T.anisopteriodeus* and *T.sinensis*. Moreover, phylogenetic analysis of combined LSU and ITS sequences places *Triscelophorus* in Microthyriaceae (Fig. 1).

*Isthmolongispora* Matsush. was established in 1971 and, so far, eleven species were accepted in this genus (Matsushima 1971; Wijayawardene et al. 2018b, 2020). In this study, ten isolates have similarity to some *Isthmolongispora* species. Of these, four isolates were identified as *Isthmolongisporaquadricellularis*, based on morphology. The combined LSU and ITS tree (Fig. 1) showed that the other six isolates formed two clades in Microthyriaceae. Comparing their morphological differences between species of the two clades, we established two new genera *Antidactylaria* and *Isthmomyces*. *Antidactylaria* includes a new species *A.minifimbriata* and a new combination *A.ampulliforma* and is phylogenetically close to two asexual species *Scolecobasidiumtropicum* Matsush. and *Neoscolecobasidiumagapanthi* Crous. *Isthmomyces* includes three new species, *I.dissimilis*, *I.oxysporus and I.macrosporus* and a new combination *I.lanceatus*. Phylogenetically, *Isthmomyces* is near to the sexual genus *Microthyrium* and the asexual genus *Neoanungitea*. Although *Ishmomyces* is closely related to *Microthyrium*, their ITS sequence similarity is low, so we cannot determine the connection between them. Based on the two-gene tree, we speculated that *Isthmolongispora* is polyphyletic. So far, at least 14 genera of aquatic hyphomycetes have shown to be polyphyletic using sequence information from a single or two genes (Nikolcheva 2002; Tsui et al. 2006; Baschien and Szewzyk 2006; Campbell et al. 2006; Duarte et al. 2015).

With increasingly widespread use of molecular techniques, multi-genes were concatenated to resolve phylogenetic affiliations and taxonomic placements at family or higher ranks. For example, SSU, LSU, *tef*1, *rpb*1 and *rpb*2 were combined to assess phylogeny (Schoch et al. 2006, 2009; Wijayawardene et al. 2014). However, sequence data and cultures of many aquatic hyphomycetes were unavailable. By 2013, over 300 aquatic hyphomycete species had been described, based on conidia morphology and conidiogenesis. However, fewer than 50 species had published ITS sequences in the International Nucleotide Sequence Database (Duarte et al. 2013). In addition, most of these species with ITS sequences were considered Ascomycota genera are *incertae sedis* because of the limitations of ITS as a phylogenetic marker for these organisms.

Molecular phylogeny of freshwater fungi in Dothideomycetes has been studied by Shearer et al. (2009) using SSU and LSU for 84 isolates representing 29 genera. The results showed that the majority of freshwater Dothideomycetes belonged to Pleosporomycetidae, including four clades comprised of only freshwater taxa, while the remaining freshwater taxa were distributed amongst other clades. In the largest phylogenetic assessment of Dothideomycetes up to 2009, members of the class from various ecological niches were included and freshwater taxa were in different clades (Schoch et al. 2009). Unfortunately, like other studies, though representative, these two studies of Dothideomycetes and freshwater ascomycetes had very few aquatic asexual genera. In the paper of Shearer et al. (2009), only 10 asexual genera were included, while in the paper of Schoch et al. (2009), only four asexual genera were included (*Monotosporella* S. Hughes and *Beverwykella* Tubaki belonging to Melanommataceae G. Winter, while *Helicomyces* Link and *Helicosporium* Nees belonging to Tubeufiaceae). Amongst the accepted genera of Dothideomycetes, only 11 aquatic or aero-aquatic asexual genera have been described as belonging to different families of the subclass Pleosporomycetidae (Wijayawardene et al. 2014). Our study provides the molecular evidence for asexual aquatic fungi.

## Conclusions

This study described two new asexual genera and six new asexual species of aquatic hyphomycetes. Our phylogenetic analyses placed several other aquatic genera in the family Microthyriaceae. Though we failed to connect teleomorphs and anamorphs at genus level, our results showed close phylogenetic relationships between aquatic hyphomycetes and Microthyriaceae at the family rank. This study also revealed the importance of obtaining pure cultures of aquatic fungi and multiple gene sequences from them to identify the origins and phylogenetic positions of aquatic hyphomycetes and their relationships with their terrestrial relatives.

## Supplementary Material

XML Treatment for Microthyriaceae

XML Treatment for Antidactylaria

XML Treatment for Antidactylaria ampulliforma

XML Treatment for Antidactylaria minifimbriata

XML Treatment for Isthmomyces

XML Treatment for Isthmomyces dissimilis

XML Treatment for Isthmomyces lanceatus

XML Treatment for Isthmomyces macrosporus

XML Treatment for Isthmomyces oxysporus

XML Treatment for Triscelophorus

XML Treatment for Triscelophorus anisopteriodeus

XML Treatment for Triscelophorus sinensis

XML Treatment for Isthmolongispora quadricellularia
